# Editorial: Modulation of Growth and Development of Tree Roots in Forest Ecosystems

**DOI:** 10.3389/fpls.2022.850163

**Published:** 2022-02-15

**Authors:** Antonio Montagnoli, Donato Chiatante, Douglas L. Godbold, Takayoshi Koike, Boris Rewald, R. Kasten Dumroese

**Affiliations:** ^1^Laboratory of Environmental and Applied Botany, Department of Biotechnology and Life Science, University of Insubria, Varese, Italy; ^2^Department of Forest and Soil Sciences, Institute of Forest Ecology, University of Natural Resources and Life Sciences (BOKU), Vienna, Austria; ^3^Department of Landscape Carbon Deposition, Global Change Research Institute, Academy of Sciences of the Czech Republic, Ceske Budejovice, Czechia; ^4^Research Faculty of Agriculture, Hokkaido University, Sapporo, Japan; ^5^Rocky Mountain Research Station, U.S. Department of Agriculture Forest Service, Moscow, ID, United States

**Keywords:** root growth, root traits, fungal community, root architecture, climate change, environmental stress, tree stability, forest dynamics

More research on the above-ground compartment of plants has been completed compared to the belowground plant compartment. Much of this disparity is likely ascribable to the methodological difficulties of studying plant roots, the associated rhizosphere, and related traits (Freschet et al., 2021a). During the past two decades, a focus on methodological advancements has rendered root investigations more feasible and more standardized in terms of quantitative approaches (Bao et al., 2018; Montagnoli et al., 2018; Atkinson et al., 2019; Chiatante et al., 2019; Cabal et al., 2021; Freschet et al., 2021b). This has allowed and encouraged a greater number of scientists to examine the hidden half of plants, thereby helping to reduce this long-standing knowledge discrepancy (Ryan et al., 2016; Freschet et al., 2021b).

These recent studies covered a wide range of root aspects, spanning from sub-cellular levels (Jung and McCouch, 2013; Wachsman et al., 2015; Slovak et al., 2016; Chiatante et al., 2021) to the community and ecosystem scales (Hopkins et al., 2013; Li et al., 2018; Freschet et al., 2021b). In association with the overall increase of root studies, scientific journals have published several thematic special issues covering diverse viewpoints, from a fundamental biological approach to identifying root phenotypes for a better understanding of responses to environmental cues. Finally, the accumulation of data regarding root system traits led to the establishment and development of databases representing local to global scales (Kattge et al., 2020; Guerrero-Ramirez et al., 2021; Iversen et al., 2021; Montagnoli et al., 2021), allowing scientists to perform reviews and meta-analyses and create new developmental models that enhance our understanding of root complexity in terms of dynamics, form, and function (Wang et al., 2018, 2019, 2021; Carmona et al., 2021).

The evidence of the effects of global climate change on vegetation in general, and on root systems in particular, continues to challenge our understanding (Norby and Jackson, 2000; Brunner et al., 2015; Zhang et al., 2019; Masyagina et al., 2021). Although forests represent an important ecosystem for reducing the impacts of climate change, climate change itself and the environmental stressors exacerbated by it (e.g., fire, drought, wind, and flooding) strongly affect forests and the services they provide (Wagner et al., 2014; Locatelli, 2016; Nordström et al., 2019). Analyzing the effects of these factors on the growth and development of single trees, and their complex organization into forest, is necessary for understanding and increasing the resilience of this ecosystem. Responses of forest ecosystem functions to climate change are strongly linked to changes in compositional and structural complexity (Ehbrecht et al., 2021). Indeed, a more complex forest ecosystem may respond differently to new and variable conditions, enhancing both ecosystem functional stability and services sustainability (Brockerhoff et al., 2017; Messier et al., 2019). This functional tree network is dependent on below-ground complexity (i.e., root plasticity and symbiotic relationship of roots with microbes such as fungi) and its integration with the above-ground structure (Freschet et al., 2021b). Unfortunately, despite more work being reported on roots, knowledge of the below-ground forest remains scarce, requiring an in-depth understanding of root function, complexity, and relationship with microbes. Thus, the 12 papers within this Frontiers in Plant Science Research Topic represent a contribution toward amplifying the knowledge regarding tree root development at the single–tree and forest levels. The aim is to reveal the impact of environmental stressors on root growth and how the sum of these impacts at the individual tree level may endanger the performance and resilience of entire forest ecosystems.

Seven manuscripts focus on the relationship between roots and water, revealing how plants plastically respond to the changing of water availability through the modulation of different root traits. In particular, Lihui et al., in regions with different precipitation regimes and slope aspects, highlighted how variations in the belowground rooting depth may be a key functional trait determining plant survival and growth in drought-prone regions. Similarly, Fujita et al., showed how potted seedlings subjected to different depths of waterlogging adapt to hypoxic conditions by changing the depth of their fine root distribution. Seedlings subjected to different drought and soil substrate conditions in pots maintained a low cost-benefit ratio in root system development under drought conditions by varying the morphological traits of different fine root branching orders and non-structural carbohydrate content (Ji et al.). In line with these findings, a forest-scale study with trees exposed to extreme drought conditions revealed that the responses of fine roots of different species are not linked to a sole ecological strategy (Nikolova, Bauerle et al.). Using a split-pot design to apply spatially heterogeneous soil moisture, Hart et al. discerned a differential mass or reserve allocation between above- and belowground organs as well as within the root system, suggesting that different portions of plant organs might respond autonomously to local conditions. A shovelomics approach within afforestation stands of arid lands showed that root biomass was correlated positively with water availability and negatively with fertilizer application (Nyam-Osor et al.). Finally, Withington et al., in a common garden experiment with different temperate tree species, suggested that annual absorptive fine-root growth response to extreme precipitation or drought events can be exacerbated across years through a “legacy effect.”

The response of roots and their microorganisms to forest management activities is another important Research Topic. Koczorski et al. in a short rotation coppice system found that the main factors controlling fungal communities were soil properties and the level of fungal community association with the trees. In a mixed stand, Veselá et al. showed how management after disturbance (i.e., windstorm) significantly affected the composition of ectomycorrhiza on a species-specific basis but did not affect the potential connections of trees via their ectomycorrhizal symbionts. Nikolova, Geyer, et al. found that subdominant trees on the edges of strip cuttings presented a strong increase of root growth relative to shoot growth that persisted 7–8 years and probably supported the need to enhance wind firmness through improving root anchorage. Differences and/or similarities between stems and roots in response to mechanical constraints were examined by De Zio et al. They found an antagonistic interaction of auxin and cytokinin signaling that was highly organ-dependent and resulted in the production of reaction wood on the stretched side of stems and on the compressed side of roots. Finally, using the ingrowth core method, Noguchi et al. found that fine root growth of understory plants was increased by Sphagnum moss. This finding is key to better understanding belowground carbon dynamics, especially in forests sites where the shallow permafrost layer varies in thickness.

Together, these 12 studies provide new evidence on how roots, rhizospheres, and microorganisms are coordinated with the aboveground tree organs into an integrated whole organism that responds and adapts to changing environmental conditions. This research offers important insights into the ecosystem function of current and future forests. Yet, however, a main challenge in tree root research is to gain a complete understanding framework of integrated responses to the constant change of environmental conditions from the molecular level upscaled to tissue, single organism, and forest community scales, the later requiring a comprehension of biological communication mediated by microorganisms. This would lead to a more detailed understanding of carbon dynamics and carbon use efficiency of the entire tree and forest.

## Author Contributions

AM conceived and produced the first draft. BR provided important insights to the first draft. DC, DG, and TK commented on the final draft. AM and RKD equally contributed to all revisions leading to the final manuscript.

## Conflict of Interest

The authors declare that the research was conducted in the absence of any commercial or financial relationships that could be construed as a potential conflict of interest.

## Publisher's Note

All claims expressed in this article are solely those of the authors and do not necessarily represent those of their affiliated organizations, or those of the publisher, the editors and the reviewers. Any product that may be evaluated in this article, or claim that may be made by its manufacturer, is not guaranteed or endorsed by the publisher.
